# Genetic spectrum of prenatally diagnosed skeletal dysplasias in a Finnish patient cohort

**DOI:** 10.1002/pd.6186

**Published:** 2022-06-02

**Authors:** Katri Rajala, Ellamaija Kasanen, Sanna Toiviainen‐Salo, Helena Valta, Outi Mäkitie, Vedran Stefanovic, Laura Tanner

**Affiliations:** ^1^ Department of Clinical Genetics Kuopio University Hospital Kuopio Finland; ^2^ University of Helsinki Helsinki Finland; ^3^ Department of Pediatric Radiology HUS Medical Imaging Center Helsinki University Hospital University of Helsinki Helsinki Finland; ^4^ Research Program for Clinical and Molecular Metabolism Faculty of Medicine University of Helsinki Helsinki Finland; ^5^ Children’s Hospital and Pediatric Research Center Helsinki University Hospital University of Helsinki Helsinki Finland; ^6^ Department of Obstetrics and Gynecology Fetomaternal Medical Center Helsinki University Hospital University of Helsinki Helsinki Finland; ^7^ HUSLAB Department of Clinical Genetics Helsinki University Hospital Helsinki Finland; ^8^ Department of Medical and Clinical Genetics University of Helsinki Helsinki Finland

## Abstract

**Objective:**

This retrospective cohort study aims to describe the genetic spectrum of fetal skeletal dysplasias detected in a Finnish patient cohort and the diagnostic yield of various analysis methods used.

**Method:**

A total of 121 pregnancies with prenatally suspected or diagnosed skeletal dysplasia were analyzed between 2013 and 2020. Clinical details and findings from genetic testing were collected.

**Results:**

Abnormal ultrasound triggered further testing in most cases. However, there were several cases with increased nuchal translucency and/or abnormal risk ratio in the first trimester combined screening as the initial finding. Further genetic testing was performed in 84/121 (69.4%) cases. A genetic diagnosis was confirmed in 36/84 (42.9%) cases. Half of the identified cases could be attributed to a founder mutation specific to the Finnish Disease Heritage, whereas the other half consisted of a variety of other genetic defects.

**Conclusion:**

In our patient cohort, the overall genetic spectrum of prenatally diagnosed skeletal dysplasias was wide. However, the impact of Finnish founder mutations was considerable, suggesting that the genetic spectrum of skeletal dysplasias may differ significantly between populations. This should be taken into consideration during the diagnostic process especially as initial ultrasound findings may be unspecific and the interpretation of ultrasound features is usually difficult.

## Key points

What's already known about this topic?

- Prenatal diagnosis of skeletal dysplasia is challenging and broader diagnostic methods have increased the diagnostic yield. In addition to well‐described ultrasound findings, increased nuchal translucency and/or abnormal first trimester combined screening could indicate skeletal disease.

What does this study add?

- We observed a considerable number of rare skeletal dysplasias enriched in the Finnish population. There may be considerable population‐specific variation in the mutation spectrum of skeletal dysplasias, which should be considered in the diagnostic process.

## 1 INTRODUCTION

Skeletal dysplasias are rare inherited disorders that disrupt the normal bone formation, growth, density, or mineralization occurring in 1/5000 births.^1^, ^2^ According to the recent (2019) Nosology and classification of genetic skeletal disorders, 461 different disorders have been recognized to date with pathogenic variants in 437 different genes.^3^

The spectrum of skeletal dysplasias in the Finnish population is different compared to other European countries. Some diseases are more prevalent in Finland while being very rare elsewhere. This spectrum of disease is called the Finnish Disease Heritage (FDH). Several bottlenecks and inhabitation of remote areas are considered to have caused the enrichment of some disease‐causing gene defects and losses of others during the course of Finland's population history. The FDH contains 36 monogenic diseases including skeletal dysplasias such as diastrophic dysplasia and cartilage‐hair hypoplasia. Albeit overrepresented, the disorders are still rare even in the Finnish population.^4^, ^5^, ^6^, ^7^, ^8^, ^9^, ^10^, ^11^ On the other hand, increasing immigration diversifies the spectrum of skeletal dysplasias observed in the Finnish population.

The most severe forms of skeletal dysplasias are associated with significant perinatal morbidity and mortality.^12^ According to the Finnish law, the termination of pregnancy (TOP) is allowed before 20 pregnancy weeks if there is considerable suspicion of a severe fetal structural or/and genetic abnormality and before 24 weeks of pregnancy if such an abnormality is diagnosed with a reliable method. Permission of the National Supervisory Authority for Welfare and Health (Valvira) is required for termination. Diagnosis of prenatal‐onset skeletal dysplasias mostly relies on ultrasound findings and is supported by MRI and/or genetic analysis from a chorionic villus biopsy or an amniotic fluid sample. Postnatally, additional evidence may be achieved from radiographs and in case of termination or fetal demise, autopsy findings.^13^, ^14^, ^15^ Diagnostic accuracy of prenatal US alone is only 40%–68%^14^, ^16^, ^17^ and other supportive methods are therefore needed.

When fetal skeletal dysplasia is suspected, the limited time window for genetic diagnostics has been a challenge. Before the emergence of NGS‐based methods, the diagnostic possibilities were limited to analyses of some common disease‐causing variants such as the recurrent *FGFR3* mutations in achondroplasia and thanatophoric dysplasia or the Finnish founder mutations for diastrophic dysplasia and cartilage‐hair hypoplasia. Next generation sequencing (NGS) allows simultaneous analysis of several genes in a shorter timeline, considerably expanding the prenatal diagnostic options. This is especially important as other genetic diseases such as arthrogryposis, some connective tissue disorders, and neurological diseases limiting fetal movements may present with findings closely resembling skeletal dysplasias during the fetal period.^18^

Most genetic analyses are time‐consuming and costly. In addition, ultrasound findings suggesting skeletal dysplasia may not be detected early enough for the specific diagnosis to be available within the legal time window for the termination of pregnancy. Interpretation of the genetic analysis may also be a challenge. Genome‐wide analyses such as whole exome sequencing or comparative genomic hybridization (array‐CGH) may also result in secondary findings raising ethical issues.^19^

The aim of this study was to analyze the genetic spectrum of skeletal dysplasias detected prenatally in a tertiary Finnish referral university hospital and the diagnostic yield of various analysis methods used in obtaining the diagnoses.

## 2 MATERIALS AND METHODS

This retrospective cohort study was performed at the Fetomaternal Medical Center (FMC) at Helsinki University Hospital. FMC is a tertiary institution for fetal medicine providing fetal diagnostics and treatment as well as genetic counseling in high‐risk pregnancies. FMC provides services for almost a third of the Finnish population. Between January 2013 and March 2020, we analyzed all cases of prenatally suspected or diagnosed cases of fetal skeletal dysplasias examined at FMC. Clinical details about the pregnancies, including ultrasonographic, pathological, and genetic findings were collected from patient records. Research permit for this study was obtained from Helsinki University Central Hospital (no. 4199, November 4, 2020). As this was a retrospective analysis, informed consent and separate ethical approval were not requested.

**FIGURE 1:**
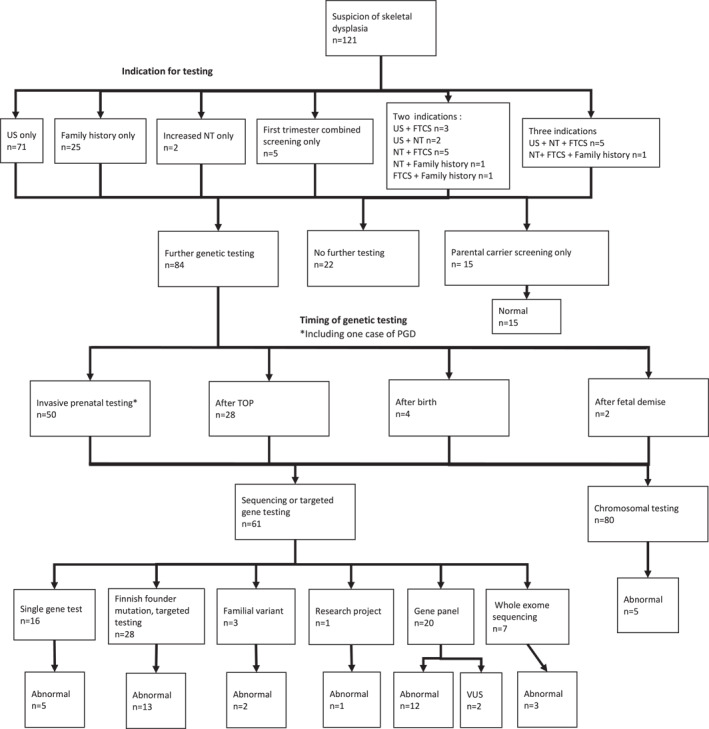
The diagnostic process and genetic findings in the 121 pregnancies with a suspicion of skeletal dysplasia. FTCS, first‐trimester combined screening; NT, nuchal translucency; US, ultrasound

In Finland, all pregnant women are offered prenatal screening to determine the risk for genetic disorders or birth defects. Screening is free of charge at local maternity clinics regardless of social or legal status. The screening program consists of first trimester combined screening between 10^+1^ and 13^+6^ weeks [general ultrasound examination and measurement of nuchal translucency (NT) combined with maternal blood tests of PAPP‐A and β‐hCG] and second‐trimester morphology ultrasound between 18^+0^ and 21^+6^ gestational weeks. Indications for further evaluation by a specialist include an abnormal screening result (≥1:250 risk for trisomy 21 or ≥1:150 risk for trisomy 18) in first‐trimester screening, structural anomalies detected with ultrasonography, increased NT or positive family history, or known carriership for an early‐onset genetic disease. Genetic counseling and NIPT or invasive diagnostic testing (either chorionic villus sampling or amniocentesis) are offered in cases with suspected genetic etiology, and if the further results would affect parental decision‐making. Non‐invasive prenatal testing for common trisomies is offered as an alternative to invasive testing in cases with a positive combined screening result and/or slightly increased NT (up to 3.4 mm). In case of fetal demise or termination of pregnancy without prior molecular genetic diagnosis, post‐mortem investigations (including fetal autopsy, imaging, and genetic analyses) may be performed to confirm the diagnosis and estimate the recurrence risk in future pregnancies. Parental carrier screening for Finnish disease heritage is not offered at the moment but targeted testing can be offered for close relatives of known carriers. The diagnostic path is depicted in Figure 1.

Laboratory methods included trisomy PCR, chromosomal analysis, and array‐CGH, which were performed in our own laboratory (HUSLAB). Trisomy PCR with Aneufast Multiplex QF‐PCR Kit was used to detect trisomies,^13^ ^,^ ^18^ ^,^ ^21^ sex chromosome abnormalities, and triploidies. Array‐CGH was performed using the Agilent Human Genome CGH Microarray Kit 180K with a resolution of 50–200 kb. Single gene tests, gene panels, and whole exome sequencing (WES) were performed with next generation sequencing (NGS) in different accredited laboratories. Maternal cell contamination testing was performed for all chorionic villus and amniotic fluid samples.

## 3 RESULTS

During the research period (7.1 years), there were approximately 15,500 deliveries per year at Hospital District of Helsinki and Uusimaa and suspicion of a skeletal dysplasia arose in 121 pregnancies of which 66 led to live birth. Abnormal ultrasound findings were the primary indicators of skeletal abnormality (*n* = 81, 66.9%). Increased NT was present in 16 cases (13.2%). First trimester combined screening indicating that the increased risk ratio for common chromosomal abnormalities was abnormal in total of 20 cases (16.5%), out of which in five cases (4.1%), it was the only abnormal finding that triggered further investigations. In these five cases, NT itself was within the reference range. Additionally, in 26 cases (20.7%), there was a positive family history for a skeletal disorder, and in 23/26 cases, the familial mutation was already known. In 4/26 cases (3.3%), there were previous pregnancies with skeletal disease, and in 2/26 cases (1.6%), a parent was known to be a carrier of an autosomal dominantly inherited skeletal disorder.

In 22/121 (18.2%) pregnancies, no invasive fetal testing was offered. These cases included either normal findings in repeated ultrasounds or fetuses with isolated or likely nongenetic abnormalities, such as clubfoot or amniotic band syndrome. In 15 pregnancies, the risk for a previously known familial mutation was ruled out by parental carrier testing and no further analyses were needed. Overall, 84 cases (69.4%) proceeded with further genetic testing.

**FIGURE 2:**
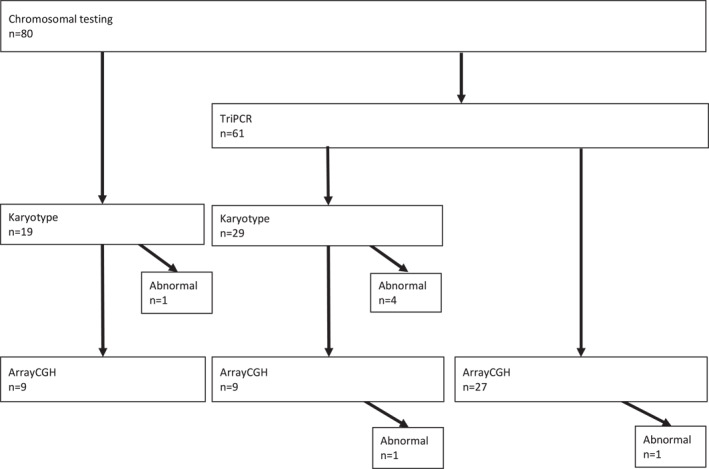
The chromosomal testing process. The figure depicts different combinations of chromosomal testing methods performed. TriPCR, trisomy PCR

Analysis for aneuploidies either by karyotyping or rapid testing for common trisomies with real‐time quantitative PCR followed by array‐CGH was performed as a first‐tier test for all fetuses as aneuploidy has been reported to be associated with abnormal skeletal development.^20^ In this cohort, three cases of trisomy 18, one case of trisomy 13, and one case of mosaic trisomy 16 combined with a balanced translocation (X; 7) were diagnosed. No cases with a copy number variant explaining a skeletal abnormality were detected. Figure 2 shows the pathway of chromosomal testing in this study.

A genetic diagnosis was obtained in 36 of the 84 cases proceeding to genetic testing (42.9%). Half of these cases (*n* = 18, 50%) belonged to the previously mentioned FDH group and a biallelic Finnish founder mutation could be identified. These diagnoses included lethal congenital contracture syndrome 1 (OMIM: 253310), diastrophic dysplasia (OMIM: 222600), cartilage‐hair hypoplasia (CHH) (OMIM: 250250), GRACILE syndrome (OMIM: 603358), hydrolethalus syndrome (OMIM: 236680), and Mulibrey nanism (OMIM: 253250). The other 50% (*n* = 18) consisted of a variety of diagnoses listed in Tables 1 and 2. Most cases had a clear genetic diagnosis with a disease‐related phenotype. Two cases of spondylocostal dysostosis 5 had mutations in TBX6 gene but the significance of the identified variants is partly unclear. Further testing was offered to the families.

**TABLE 1:** Genetically confirmed cases of Finnish disease heritage

Case	Diagnosis	Phenotype	Ultrasound screening	Sequencing result	Genotype	Mutation type	Classification
1	Diastrophic dysplasia	Short lower limbs with abnormal position	H12+0	SLC26A2 c.‐26+2T>C (IVS1 ds T‐C +2) NM_000112.3	HOM, AR	Splicing mutation	Pathogenic Finnish founder mutation ClinVar ID:4097
2	Diastrophic dysplasia	Short long bones, abnormal ankle position, knees bent	H12+4	SLC26A2 c.‐26+2T>C (IVS1 ds T‐C +2) NM_000112.3	HOM, AR	Splicing mutation	Pathogenic Finnish founder mutation ClinVar ID:4097
3	Diastrophic dysplasia	Short upper and lower limbs. Femur and humerus 4–5 weeks smaller. Abnormal position of ankles and wrists	H21+0	SLC26A2 c.‐26+2T>C (IVS1 ds T‐C +2) NM_000112.3	HOM, AR	Splicing mutation	Pathogenic Finnish founder mutation ClinVar ID:4097
4	Diastrophic dysplasia	Second trimester US: Short long bones and some of them bowed, suspected small chest	H13+5	SLC26A2 c.‐26+2T>C (IVS1 ds T‐C +2) NM_000112.3	HOM, AR	Splicing mutation	Pathogenic Finnish founder mutation ClinVar ID:4097
5	Lethal congenital contracture syndrome‐1	Fetus not moving. H13+1 US: Limbs not moving, lower limbs straight, upper limb flexed, feet turned inwards. Artrogryposis suspected	H12+1	GLE1 c.433‐10A>G NM_001003722.1 NG_012073.1	HOM, AR	Splicing mutation	Pathogenic Finnish founder mutation ClinVar ID:6462
6	Lethal congenital contracture syndrome‐1	Widespread hydrops on back, upper limbs flexed in front of the body, ulnardeviation on wrists, lower limbs straight and in 90° angle. No movements	H13+3	GLE1 c.433‐10A>G NM_001003722.1 NG_012073.1	HOM, AR	Splicing mutation	Pathogenic Finnish founder mutation ClinVar ID:6462
7	Lethal congenital contracture syndrome‐1	No fetal movements. Lower limbs straight, pleural and pericardial effusion. Club foot and fetal hydrops. NT 2.6–2.9 mm	H11+6	GLE1 c.433‐10A>G NM_001003722.1 NG_012073.1	HOM, AR	Splicing mutation	Pathogenic Finnish founder mutation ClinVar ID:6462
8	Lethal congenital contracture syndrome‐1	No fetal movements. NT 6.4 mm. Humerus and femur shorter. fetal hydrops. Lower limbs straight and upper limbs flexed	H14+5	GLE1 c.433‐10A>G NM_001003722.1 NG_012073.1	HOM, AR	Splicing mutation	Pathogenic Finnish founder mutation ClinVar ID:6462
9	Lethal congenital contracture syndrome‐1	No fetal movements. Long bones short, fetal hydrops. Upper limbs flexed, fingers in ulnar deviation. Knees hyperextended and hips flexed. Club foot	H20+1	GLE1 c.433‐10A>G NM_001003722.1 NG_012073.1	HOM, AR	Splicing mutation	Pathogenic Finnish founder mutation ClinVar ID:6462
10	Lethal congenital contracture syndrome‐1	NT 9.5 mm, fetal hydrops. Upper limbs in malposition and suspected bilateral club foot	H13+2	GLE1 c.433‐10A>G NM_001003722.1 NG_012073.1	HOM, AR	Splicing mutation	Pathogenic Finnish founder mutation ClinVar ID:6462
11	Lethal congenital contracture syndrome‐1	No fetal movements in first US. H18+0 US: Upper and lower limb contractures. Hips flexed and knees straight. Ankles and wrists in malposition, micrognathia	H17+3	GLE1 c.433‐10A>G NM_001003722.1 NG_012073.1	HOM, AR	Splicing mutation	Pathogenic Finnish founder mutation ClinVar ID:6462
12	Cartilage‐hair hypoplasia (di‐di twin pregnancy, both fetuses affected)	Both fetuses: Short femurs (4 weeks smaller). Also ulnae, radiae, fibulae, tibiae and feet short	H21+4	Both fetuses: RMRP n.71A>G NG_017041.1 NR_003051.3	HOM, AR	Non‐coding transcript variant	Pathogenic Finnish founder mutation ClinVar ID:14208
13	Cartilage‐hair hypoplasia	Humerus and femur 2 weeks smaller	H20+4	RMRP n.71A>G NG_017041.1 NR_003051.3	HOM, AR	Non‐coding transcript variant	Pathogenic Finnish founder mutation ClinVar ID:14208
14	GRACILE syndrome	Long bones short and head small. Dilated colon	H20+4	BCS1L c. 232A>G p.(Ser78Gly) NM_001079866.2	HOM, AR	Missense	Pathogenic Finnish founder mutation ClinVar ID:6167
15	GRACILE syndrome	Small fetus, humerus and femur short, dilated bowels	H20+5	BCS1L c. 232A>G p.(Ser78Gly) NM_001079866.2	HOM, AR	Missense	Pathogenic Finnish founder mutation ClinVar ID:6167
16	Hydrolethalus syndrome	NT 3.5 mm, hygroma. Abnormal brain structure, suspected Dandy‐Walker anomaly. Dilated fourth ventricle. Adnormally shaped face, absent nasal bone. Midline anomaly	H13+5	HYLS1 c.632A>G p.(Asp211Gly) NM_145014.3	HOM, AR	Missense	Pathogenic Finnish founder mutation HGMD: CM056966
17	Hydrolethalus syndrome	Short limbs, large bladder, ankles inverted, suspected cleft lip	H12+0	HYLS1 c.632A>G p.(Asp211Gly) NM_145014.3	HOM, AR	Missense	Pathogenic Finnish founder mutation HGMD: CM056966
18	Mulibrey nanism	Asymmetric growth disorder	H30+0	TRIM37 c.493‐2A>G NM_015294.6	HOM, AR	Splicing mutation	Pathogenic Finnish founder mutation HGMD: CS001838

Abbreviations: AD, autosomal dominant; AR, autosomal recessive; FMC, Fetomaternal Medical Center; HET, heterozygote; HOM, homozygote; US, ultrasound; VUS, variant of unknown significant.

**TABLE 2:** Other genetically confirmed skeletal dysplasias

Case	Diagnosis	Phenotype	Ultrasound screening	Sequencing result	Genotype	Mutation type	Classification
1	Osteogenesis imperfecta type IIA	Short limbs, femur 5 weeks smaller. Thorax bell‐shaped, suspected pulmonar hypoplasia. Low mineralization in skull	H20+6	COL1A1 c.1353+6T>G NM_000088.3	HET, AD	Splicing mutation	Likely pathogenic. Not previously reported in literature. Variant next to it is raported pathogenic and leads to splicing error
2	Osteogenesis imperfecta type I	No fetal anomalies, bones looked normal in US. After birth: Blue scleras, no fractures. At the age of 3 fractured toe after a fall	H20+4	COL1A1 c.103+1G>A (IVS1+1G>A) NM_000088.3 NG_007400.1	HET, AD	Splicing mutation	Likely pathogenic HGMD: CS042148
3	Osteogenesis imperfecta type IIA	Broken ribs, abnormal shape in chest, short femurs (7 weeks smaller) and other bowing. Short tibiae and other bowing. Club‐foot on right. Upper limbs short and bowing	H21+2	COL1A1 c.1922G>A p.(G641E) NM_000088.3	HET, AD	Missense	Likely pathogenic not reported in HGMD or ClinVar
4	Osteogenesis imperfecta type III	Femur ja humerus over 2 weeks behind	H21+5	COL1A1 c.2461G>A, p.(Gly821Ser) NM_000088.3	HET, AD	Missense	Pathogenic ClinVar ID:425610
5	Osteogenesis imperfecta type III	US: Short long bones, bowing femurs, possibly narrow thorax	H20+2	COL1A1 c.1777G>A p.(Gly593Ser) NM_000088.3	HET, AD	Missense (confirmed by Sanger)	Pathogenic ClinVar ID:17326
6	Osteogenesis imperfecta type III	Short femur and humerus. Tibia and fibula short and bowing	H20+1	COL1A2 c.3034G>A p.(Gly1012Ser) NM_000089.3	HET, AD	Missense	Pathogenic ClinVar ID:216908
7	Osteogenesis imperfecta type III	Very short limbs, both humeri extremely short, both humerus and femus bowing, poor ossification, most skull bones missing, lower limbs dysplastic, parts of tibia missing, narrow bell‐shaped thorax, short ribs	H20+4	COL1A2 c. 1971+1G>C NM_000089.3	HET, AD	Splicing mutation	Likely pathogenic not reported in HGMD or ClinVar
8	Tanatophoric dysplasia	All long bones short, femur bowing and lenght equated H15+1. Head circumference H21+5. Slightly small chest	H20+0	FGFR3 c.742C>T p.(Arg248Cys) NM_000142.4	HET, AD	Missense	Pathogenic ClinVar ID:16332
9	Tanatophoric dysplasia	Short femur and humerus. H13+6 US: Humerus and femur bowing, narrow thorax. Tanatophoric dysplasia suspected	H12+6	FGFR3 c.746C>G p.(Ser249Cys) NM_000142.4	HET, AD	Missense	Pathogenic ClinVar ID:16339
10	Spondylocostal dysostosis 5	Fetal hydrops	H11+2	TBX6 c.484G>A p.(Gly162Ser); 815G>A p.(Arg272Gln); c.622‐2A>T	Compound HET, AD/AR	Missense, splicing mutation	484G>A: HGMD (CM1313171) ClinVar ID:786429 benign; 815G>A: HGMD (CM1313172), ClinVar ID734843 benign; 622‐2A>T: HGMD (CS1313170), not reported in ClinVar
11	Spondylocostal dysostosis 5	Fetal hydrops, short trunk, round abdomen, stiff lower limbs	H11+2	TBX6 c.484G>A p.(Gly162Ser); 815G>A p.(Arg272Gln); c.622‐2A>T	Compound HET, AD/AR	Missense and splicing mutation	484G>A: HGMD (CM1313171) ClinVar ID: 786429 benign; 815G>A: HGMD (CM1313172), ClinVar ID734843 benign; 622‐2A>T: HGMD (CS1313170), not reported in ClinVar
12	Spondyloepifyseal dysplasia congenita	Femur and humerus 3 weeks smaller	H21+1	COL2A1 c.1196G>T p.(Gly399Val) NM_001844.4	HET, AD	Missense	Pathogenic HGMD: CM1513371
13	Spondyloepifyseal dysplasia congenita	H31+6: All long bones short, femur 3 weeks smaller	H27+5	COL2A1 c.2536G>A p.(Gly846Arg) NM_001844.4	HET, AD	Missense	Likely pathogenic ClinVar ID:280926
14	Osteochondrodysplasia congenita. Variable phenotype in heterozygotes, reduced penetrance. Fetal findigs indicates resessively inherited disease.	Second trimester US: Long bones 3 weeks shorter, chest slightly small	H12+6	ACAN c.454+5G>A and c.2227del p.(Glu743Asnfs*67) NM_013227.3	Compound HET, AD/AR	Splicing mutation and nonsense	VUS and likely pathogenic not reported in HGMD or ClinVar
15	Osteochondrodysplasia congenita	Lower limbs in malposition, narrow thorax. H15+6 US: Small chest, long bones short, right femur bowing, bilateral club foot	H13+6	COL2A1 c.2473G>A p.(Gly825Arg) NM_001844.4	HET, AD	Missense	Likely pathogenic not reported in HGMD or ClinVar
16	Short‐rib thoracic dysplasia type 3	Bowing and slightly short femur, malposition of lower limbs. H17+6 US: Long bones in lower limbs short, femurs bowing. H20 US similar findings and narrow chest.	H15+6	DYNC2H1 c.9044A>G p.(Asp3015Gly) and 244C>T p.(Arg82*) NM_001080463.1	Compound HET, AR	Missense and nonsense	Pathogenic and likely pathogenic 9044A>G: ClinVar ID:6503; 244C>T: Not reported in HGMD or ClinVar
17	Freeman‐Sheldon syndrome	Lower limbs straight and not moving. Other wrist bended and fingers kinked	H20+4	MYH3 c.2015G>A p.(Arg672His) NM_002470.3	HET, AD	Missense	Pathogenic ClinVar ID:14138
18	Type A1 brachydactyly	Long bones short (less than 3 percentile), also tibia and fibulae short	H20+5	IHH c.271T>C p.(Phe91Leu) NM_002181.3	HET, AD	Missense	Significant VUS not reported in HGMD or ClinVar. Not previously reported in literature, predicted to be harmful in 2/3

Abbreviations: AD, autosomal dominant; AR, autosomal recessive; FMC, Fetomaternal Medical Center; HET, heterozygote; HOM, homozygote; US, ultrasound; VUS, variant of unknown significant.

Genetic diseases associated with US abnormalities observed in early pregnancy (between 10 and 14 weeks of pregnancy) included diastrophic dysplasia (*n* = 3), osteogenesis imperfecta type III (*n* = 1), thanatophoric dysplasia (*n* = 1), lethal congenital contracture syndrome (*n* = 4), spondylocostal dysostosis (*n* = 2), and hydrolethalus syndrome (*n* = 2).

Of the 36 genetically confirmed cases, seven had abnormal first trimester screening results (≥1:250). Nuchal translucency (NT ≥ 3.0 mm) was increased in seven cases. In three cases, both NT and screening results were abnormal.

**FIGURE 3:**
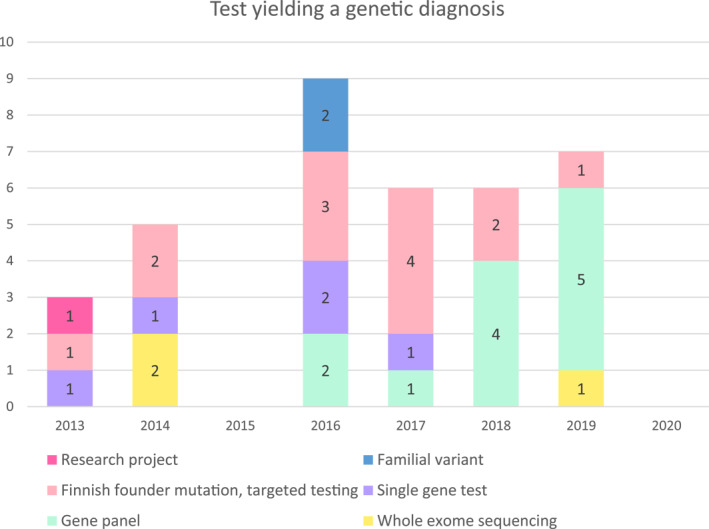
Genetic testing methods leading to a confirmed diagnosis of a skeletal dysplasia. Figures represent the number of diagnoses made using the method in question

In the cases associated with the FDH, the diagnosis was reached in 72.2% (*n* = 13) by targeted testing of the Finnish founder mutation, in two cases (11.1%) with a comprehensive gene panel, in two cases (11.1%) with sequencing of a single gene, and in one case (5.6%) with WES. In the remaining cases (*n* = 18), gene panels yielded a diagnosis in 55.6% (*n* = 10). In three cases (16.7%), the diagnoses were obtained with a single gene test, two cases (11.1%) with familial variant testing, and two cases (11.1%) with WES. In one case (5.6%), the result was obtained in a research project. This result was verified in our own laboratory. Figure 3 presents genetic testing methods leading to a confirmed diagnosis.

**FIGURE 4:**
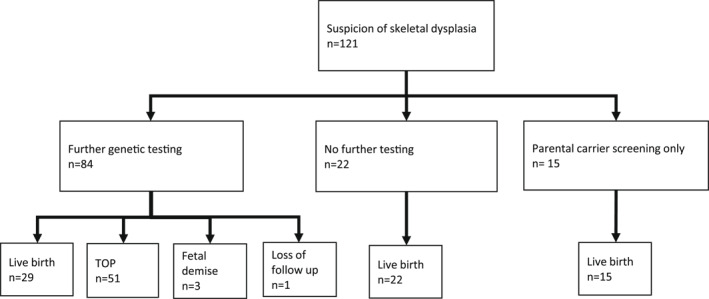
Outcome of 121 pregnancies with suspected skeletal dysplasia. TOP, termination of pregnancy

Of the pregnancies with a genetic diagnosis, nine (25%) led to a live birth while the rest were terminated. At the time of termination, a genetically confirmed diagnosis was available only in five cases (13.9%). Regarding the nine nonterminated pregnancies, in two cases, the diagnosis was reached after the legal time limit for termination had passed at 27^+5^ and 30 weeks. In one case, the OI diagnosis of the expectant mother was confirmed during pregnancy, but further fetal testing was not performed until birth. In the remaining 6 cases, the abnormalities were observed in the second trimester ultrasound. In a case of type 1 brachydactyly, termination was not indicated based on the diagnosis. In one case of CHH, one case of spondyloepiphyseal dysplasia, two severe cases of OI, and one case of GRACILE, the family decided to continue with the pregnancy despite the diagnosis. The course of pregnancies is depicted in Figure 4. The phenotype of the live‐born children was in all nine cases in line with the prenatal genetic diagnosis.

Furthermore, there were 25 cases with suspected skeletal disease in which a genetic diagnosis was not obtained during the study period. This group was very heterogeneous. In most of these cases (12/25), fetuses had multiple anomalies with skeletal involvement. In 7/25 cases, only one skeletal abnormality such as fibular hemimelia was observed. Four fetuses were suspected of having arthrogryposis. Two cases had ultrasound findings consistent with a particular type of skeletal dysplasia, but a genetic diagnosis could not be confirmed.

In two cases, a variant of unknown significance (VUS) was found with genetic testing. In the first case, the fetus had radius aplasia and an absent thumb. The gene panel found a heterozygous variant in the *CENPJ gene* classified as VUS. The pathogenic *CENPJ* gene variants have mainly been reported in individuals with microcephaly and its possible association with the fetal findings in this case remains unclear. In the second case, short long bones (humeri, femuri, tibiae, and fibulae) were observed in the second trimester US. A heterozygous variant classified as VUS in the *FLNB* gene was found with a gene panel. The same variant was also present in the asymptomatic mother.

## 4 DISCUSSION

A broad spectrum of genetic, focal, and etiologically unspecified conditions was diagnosed in fetuses initially suspected of having skeletal dysplasia. In pregnancies eventually diagnosed with genetically confirmed skeletal dysplasia, the diagnostic process could be triggered by a variety of reasons including elevated NT and/or first trimester combined screening, family history, diagnosed or suspected skeletal anomalies in previous pregnancies, or abnormal ultrasound findings in the ongoing pregnancy.

Abnormal US findings were the principal indication for further testing in all the cases in our cohort. However, 10 of the 36 fetuses with a confirmed genetic diagnosis had normal US findings at the beginning of the diagnostic process.

Increased NT was one of the primary findings in eight cases with genetically confirmed skeletal dysplasia. Interestingly, in one of these cases, the primary finding was increased nuchal translucency only. In addition, there were three cases with increased NT combined with abnormal US findings. Two cases had elevated NT and positive family history for skeletal abnormalities. Our observations were therefore consistent with previous reports describing increased NT in association with skeletal dysplasias.^21^, ^22^

In one case, with diastrophic dysplasia, with very typical US findings, the specific diagnosis could be obtained as early as at 13 pregnancy weeks. In most cases, however, the diagnostic journey was slow and distinguishing between lethal and nonlethal conditions proved difficult. We have not received any information about misdiagnoses of skeletal dysplasias. However, ultrasound findings of some skeletal diseases, such as hypochondroplasia, may appear later in pregnancy (or after birth) and prenatal diagnosis might therefore not be possible.

Termination of the pregnancy due to skeletal dysplasia (*n* = 27) took place between 12^+5^ and 24^+0^ gestational weeks. Almost half of the terminations (*n* = 13) were performed between weeks 20^+4^ and 24^+0^, which marks the legal limit for abortion in Finland. This highlights the fact that in many cases, the suspicion of skeletal dysplasia arises rather late in pregnancy and depending on the local protocol, it may be challenging to obtain the exact diagnosis within the time limit for the termination of pregnancy. In 14 cases, the first abnormal findings were detected only after 18 weeks of pregnancy. Furthermore, reaching a genetic diagnosis or sufficient certainty about the degree of severity of the phenotype for decision‐making is time‐consuming. This can also be seen in the low number of confirmed diagnoses, among all the terminated pregnancies, obtained by the time of termination (5/51, 9.8%).

In earlier years of the study period, genetic diagnostic methods available during pregnancy consisted mostly of karyotype and CMA analysis and testing for a handful of most common mutations. Gene panels and even broader diagnostic methods like WES have now become available for fetal diagnostics. The main reason for the increased usage of broader diagnostic tests is the fact that the tests have become more affordable and results are available in shorter periods of time. In fetal skeletal dysplasias, gene panels have been especially useful in increasing the diagnostic yield.^23^, ^24^, ^25^ If diagnosis does not seem clear or symptoms could indicate many different diseases, WES might be a diagnostic method of choice.^26^, ^27^

The Finnish founder mutations made up a significant proportion of the genetically confirmed skeletal dysplasias (*n* = 18, 50%) in our Finnish cohort. Hence the diagnostic yield is moderately good within this group even with a more targeted approach. On the other hand, these mutations might not always be included in the NGS panels offered by international laboratories. Therefore, information about the parents' ethnicity still holds its relevance and expertise of clinical geneticists is needed in determining appropriate tests.

The other 50% of those with a genetically confirmed diagnosis of skeletal dysplasia included relatively common diagnoses such as osteogenesis imperfecta and thanatophoric dysplasia as well as very rare entities that are challenging to diagnose with ultrasound alone. In the majority of these cases, the diagnosis was obtained either with a comprehensive gene panel or WES. It is also meaningful to observe the relationship between actual skeletal dysplasias and other conditions with a phenotype mimicking them such as lethal congenital contracture syndrome (LCCS) and Freeman–Sheldon syndrome.^28^, ^29^, ^30^

The strength of the study was the fact that it was performed in a large tertiary center. Furthermore, the diagnostic process is free of charge and therefore, financial factors do not restrict participation in further prenatal testing and leading to an overall high participation in diagnostic evaluations. We therefore have reason to believe that the results are representative and reliably portray the genetic spectrum of the population. We can conclude that the spectrum of skeletal dysplasias in Finland is largely shaped by the FDH but also globally appearing dysplasias are seen along with rare diagnoses. The spectrum of the diagnoses that do not belong to the FDH is quite similar to that previously reported in literature.^20^, ^31^, ^32^, ^33^, ^34^ Some skeletal dysplasias, such as achondroplasia, are often diagnosed later in pregnancy.^35^ Therefore, in these skeletal dysplasias, prenatal diagnostic methods are rarely of use in the management of pregnancy.

On the other hand, the evolving diagnostic methods posed a challenge to the study as different approaches were taken during the study period. NGS panels were introduced to routine practice in 2015 and the layout of the panels and the variants included are constantly under change. We also acknowledge, that practices and regulations regarding prenatal diagnostics and pregnancy termination vary in different countries, and therefore, our approach and results may not be applicable to other countries.

It is also noteworthy to examine the cases in which a genetic diagnosis was not obtained. It is undetermined whether these cases represent genetic syndromes yet to be discovered or if the phenotypes are the result of various genetic and nongenetic factors. It is also possible that the evolved testing methods could have led to accurate diagnoses of the unresolved cases in the earlier years of the study in which more limited testing was performed. Hence, further research in this field is required.

## 5 CONCLUSION

In this study, we have reported a representative cohort of prenatally presenting skeletal dysplasias in the Finnish population. There were many primary findings that triggered the diagnostic cascade. In addition to characteristic ultrasound findings, abnormal first trimester screening and increased NT could also be the triggers for further testing. The diagnoses observed in our study population included a considerable number of rare skeletal dysplasias enriched in the Finnish population. There may be considerable population‐specific variation in the mutation spectrum of skeletal dysplasias, which should be considered in the diagnostic process. Hence, it is important that these common founder variants are covered in the diagnostic tests, for example, in gene panels, when ethnic Finnish couples are involved. Suspicion of skeletal dysplasia rose late in pregnancy, so there is a need for prompt and accurate diagnostic methods.

Although abnormal ultrasound findings of the skeletal system were the trigger for further investigations in the majority of cases in our cohort, we advocate that in pregnancies with abnormal combined first trimester screening or/and increased NT thickness as the only abnormal finding(s), the awareness of the possible bone dysplasia should be always kept in mind and meticulous assessment of fetal skeletal system performed.

## CONFLICT OF INTEREST

The authors declared that they have no conflicts of interest to this work.

## Data Availability

The data that support the findings of this study are available from the corresponding author upon reasonable request.
